# Genetic variants of *Anaplasma phagocytophilum *from 14 equine granulocytic anaplasmosis cases

**DOI:** 10.1186/1756-3305-4-161

**Published:** 2011-08-16

**Authors:** Cornelia Silaghi, Gabriele Liebisch, Kurt Pfister

**Affiliations:** 1 Comparative Tropical Medicine and Parasitology, Faculty of Veterinary Medicine, Ludwig-Maximilians-Universität, Leopoldstr. 5, 80802 Munich, Germany; 2 Zecklab, Up'n Kampe 3, 30938 Burgwedel, Germany

## Abstract

**Background:**

Equine Granulocytic Anaplasmosis (EGA) is caused by *Anaplasma phagocytophilum*, a tick-transmitted, obligate intracellular bacterium. In Europe, it is transmitted by *Ixodes ricinus*. A large number of genetic variants of *A. phagocytophilum *circulate in nature and have been found in ticks and different animals. Attempts have been made to assign certain genetic variants to certain host species or pathologies, but have not been successful so far. The purpose of this study was to investigate the causing agent *A. phagocytophilum *of 14 cases of EGA in naturally infected horses with molecular methods on the basis of 4 partial genes (*16S rRNA*, *groEL*, *msp2*, and *msp4*).

**Results:**

All DNA extracts of EDTA-blood samples of the horses gave bands of the correct nucleotide size in all four genotyping PCRs. Sequence analysis revealed 4 different variants in the partial *16S rRNA*, *groEL *gene and *msp2 *genes, and 3 in the *msp4 *gene. One *16S rRNA *gene variant involved in 11 of the 14 cases was identical to the "prototype" variant causing disease in humans in the amplified part [GenBank: U02521]. Phylogenetic analysis revealed as expected for the *groEL *gene that sequences from horses clustered separately from roe deer. Sequences of the partial *msp2 *gene from this study formed a separate cluster from ruminant variants in Europe and from all US variants.

**Conclusions:**

The results show that more than one variant of *A. phagocytophilum *seems to be involved in EGA in Germany. The comparative genetic analysis of the variants involved points towards different natural cycles in the epidemiology of *A. phagocytophilum*, possibly involving different reservoir hosts or host adaptation, rather than a strict species separation.

## Background

Equine Granulocytic Anaplasmosis (EGA) is caused by *Anaplasma phagocytophilum*, a tick-transmitted, obligate intracellular bacterium. The vectors are ticks of the genus *Ixodes*; the main vector in Europe is *I. ricinus*, in North America *I. pacificus *and *I. scapularis*. As no transovarial transmission has been shown, a reservoir animal is necessary for the maintenance in nature [1]. Before 2001, when a reclassification based on *16S rRNA *gene similarities was proposed, the causing agent of EGA was known as *Ehrlichia equi*, which was part of the *E. phagocytophila *group (including also *E. phagocytophila*, the causing agent of tick-borne fever of ruminants, and the Human Granulocytic Anaplasmosis (HGA) - agent [2]). Different partial *16S rRNA *gene variants of *A. phagocytophilum *have been detected in previous studies from various host animals and ticks [3-7].

The first case of EGA was reported from northern California in 1969 [8]. Since then reports came from North America and different European countries [9-17]. EGA is an acute febrile disease with an average incubation period of 1-2 weeks. Clinical signs include high fever, depression, anorexia, ataxia, icterus and a lower limb oedema; laboratory findings include thrombocytopenia, anaemia and leucopenia. These abnormalities have been shown both in natural and experimental infections [14,18,19]. The administration of tetracyclines is known as an effective treatment [20], but experimental infection has demonstrated that horses can make a full recovery without treatment [21]. This may account for the relatively high seropositivity as reported in horses in Italy (17.03%) and Denmark (22.3%) [22,23]. No significant difference was found in the seroprevalence between horses with or without suspicion of vector-borne diseases, which suggests the occurrence of subclinical infections [22]. Persistence of *A. phagocytophilum *was demonstrated in the bloodstream of horses after experimental infection and was not related to clinical disease, raising the question about persistency of a natural infection after an acute outbreak [21]. The existence of persistent infection with a cyclic bacteraemia has also been demonstrated in lambs after experimental infection [24]. The epidemiology of *A. phagocytophilum *differs between the continents: the outcome of disease in humans is more severe in the US, whereas disease in cattle is virtually non-existent; by contrast, disease is severe in cattle and sheep in Europe, but much rarer and milder in humans [1]. The *A. phagocytophilum *variant pathogenic for cattle (formerly *E. phagocytophila*) caused a seroconversion in horses without clinical disease [25]. By contrast, experimental infection with the HGA-agent caused a clinical disease in horses, undistinguishable from EGA [26] and even the death of a horse in another experimental study [27]. Genetic variations have been found previously on basis of the *16S rRNA*, *groEL*, *msp2*, *msp4 *and *ankA *gene in *A. phagocytophilum *from ticks and animals, however it was not possible to attribute genetic variants to a certain host or geographic origin [3,5-7,28-30]. These investigations showed that *A. phagocytophilum *seems to show certain genetic heterogeneity; because of this we hypothesised that genetic heterogeneity would also exist in *A. phagocytophilum *naturally infecting horses and causing EGA. Several steps were taken: from 14 naturally infected horses with clinical EGA, first the *16S rRNA *gene was partially amplified and sequenced to gain a general overview of the genetic variety involved. To further analyse the variants involved in equine infections, partial *groEL*, *msp2*, and *msp4 *genes were amplified and sequenced. More than one partial *16S rRNA *gene variant was involved in equine infection and genetic variations were also detected in the other amplified parts of genes.

## Results

All 14 samples were confirmed positive for *A. phagocytophilum *by using real-time PCR. The clinical data available for these cases corresponded to the typical clinical signs in EGA [19]. All four genotyping PCRs gave bands of the correct nucleotide size for the partial genes (*16S rRNA*, *groEL*, *msp2 *and *msp4*). The sequences obtained from this study have been deposited in GenBank [GenBank: JF893886-JF893940, JF907577]. The nucleotide differences are shown in Table 1.

**Table 1:** Nucleotide differences of *Anaplasma phagocytophilum *in the amplified partial genes.

Gene	variant^e^	Nucleotide position^h^												
		**76**	**77**	**84**	**125**	**222**	**376**							
									
*16S rRNA*^a^	**A**	A	A	G	G	A	A							
	**B**	A	A	G	G	A	G							
	**D**	G	A	A	A	G	G							
	**S**	A	G	G	G	A	G							
	**X^f^**	G	A	A	G	A	G							
	**Y^g^**	G	A	G	G	A	G							

		**660**	**840**	**933**	**1013**									
											
*groEL*^b^	**a**	C	C	C	A									
	**b**	T	C	C	A									
	**r**	C	T	C	A									
	**l**	C	T	T	G									

		**158**	**136**	**277**	**288**	**376**	**393**	**917**	**927**	**939**	**943**	**966**		
				
*Msp2*^c^	**I**	**G**	**A**	**A**	G	**A**	T	A	A	G	**T**	G		
	**II**	**G**	**G**	**A**	G	**A**	T	A	A	G	**T**	G		
	**III**	**G**	**G**	**A**	G	**A**	T	G	G	T	**G**	A		
	**IV**	**A**	**G**	**G**	A	**G**	G	G	G	T	**G**	A		

		**375**	**390**	**405**	**411**	**423**	**427**	**507**	**510**	**516**	**588**	**618**	**672**	**678**
		
Msp4^d^	***c***	T	A	C	A	G	**G**	C	T	C	T	A	A	C
	***d***	C	G	T	G	G	**A**	C	T	C	T	C	A	C
	***s***	C	G	T	G	A	**G**	T	C	T	C	C	G	T

*Anaplasma phagocytophilum HZ *complete genome [NC_007797] was used as reference strain: Nucleotide positions indicate the relative position to the genes:

^a ^1,433 bp of rrsA 16S ribosomal RNA [Gene ID: 3930754],

^b ^1,653 bp of groEL chaperonin GroEL [Gene ID: 3930333],

^c ^1,098 bp of APH_1361 major surface protein 2 [Gene ID: 3930122] and

^d ^849 bp of msp4 major surface protein 4 [Gene ID: 3930710]

^e ^Not official nomenclature: It has been used in the same manner in another publication (7), the letters here correspond to the ones from that publication. *16S rRNA *gene types are shown in upper case letters; *groEL *gene types in lower case letters; *msp4 *gene types in lower case letters in italics; *msp2 *gene types in Roman numbers.

^f ^GenBank: [FJ812403]

^g ^GenBank: [FJ812406]

^h ^non-synonymous substitutions are highlighted in bold.

### *16S rRNA *gene sequences

Sequence analysis revealed 4 types in the partial *16S rRNA *gene (497 bp; here called "A" (n = 1), "B" (n = 11), "D" (n = 1), "S" (n = 1)). The *16S rRNA *gene variant "B" involved in 11 of the 14 cases was identical to the "prototype" variant causing disease in humans in the amplified part [GenBank: U02521]. The partial *16S rRNA *gene sequences from this study had 99% to 100% similarity to each other and to the *A. phagocytophilum *strain HZ [GenBank: NC_007797].

### *GroEL *gene sequences

Four variants were also found in the partial *groEL *gene (530 bp; here called "a" (n = 1), "b" (n = 11), "r" (n = 1), "l" (n = 1)). The partial *groEL *gene sequences from this study had 99% to 100% similarity on basis of nucleotides to each other and 98% to 99% to the *A. phagocytophilum *strain HZ [GenBank: NC_007797]. These nucleotide changes in the partial *groEL *gene did not result in amino acid changes.

### *Msp2 *gene sequences

The partial *msp2 *gene revealed also 4 variants (893 bp; here called "I" (n = 4), "II" (n = 7), "III" (n = 2), "IV" (n = 1)). The two main *msp2 *gene variants "I" and "II" only differed in a single nucleotide polymorphism in the amplified part (nucleotide position 136), which resulted in one amino acid exchange. *Msp2 *variant "III" had five nucleotide differences from "II" (resulting in one amino acid difference) and 6 nucleotide differences from "I" (resulting in two amino acid differences). Type "IV" had 5 nucleotide differences from "III", 10 nucleotide differences from "II" and 11 nucleotide differences from "I", resulting in 3, 4 and 5 amino acid differences, respectively (Table 1). On basis of nucleotides, all partial *msp2 *gene sequences from this study had 98% to 100% similarity amongst each other and 92% to 93% similarity to the *A. phagocytophilum *strain HZ [NC_007797]. On basis of amino acids, the similarity was 98% to 100% for sequences from this study amongst each other and 87% to 88% to the *A. phagocytophilum *strain HZ [GenBank: NC_007797].

### *Msp4 *gene sequences

Three variants were detected in the partial *msp4 *gene (343 bp; here called "*c*" (n = 11), "*s*" (n = 1) and "*d*" (n = 1)). For one sequence only 306 bp could be evaluated. The partial 343 bp *msp4 *gene sequences from this study had 96% to 100% similarity on basis of nucleotides amongst each other and 96% to 97% to the *A. phagocytophilum *strain HZ [GenBank: NC_007797]. The nucleotide difference at position 427 in variant "*d*" resulted in one amino acid exchange (Table 1).

For the 11 *A. phagocytophilum *samples from horses with the *16S rRNA *gene variant "B", the *msp4 ("variant c") *and *groEL *(variant "b") gene variant were identical, whereas on the basis of the *msp2 *it was possible to distinguish two different types. The remaining three samples were different from each other in the partial analysed genes and from the other 11 samples (Table 2).

**Table 2:** Details of the horses included in this study and the respective partial genes of *Anaplasma phagocytophilum *that were detected in them.

				Gene variants^c^			
				
Breed	**Age**^**a**^	**Sex**^**b**^	Month and year of diagnosis	*16S rRNA*	*groEL*	*Msp2*	*Msp4*
Pony	20	Mc	Jul 2007	B	b	I	*c*
Hanoverenian	n. k.	F	Oct 2007	B	b	I	*c*
Warmblood	6	Mc	Apr 2004	B	b	I	*c*
Standardbred	8	Mc	Jul 2004	B	b	I	*c*
				
Hanoverenian	13	F	May 2006	B	b	II	*c*
Westphalian Warmblood	11	Mc	Oct 2007	B	b	II	*c*
Pony	19	Mc	May 2008	B	b	II	*c*
English Thoroughbred	15	F	Jun 2008	B	b	II	*c*
Icelandic horse	9	Mc	Jun 2009	B	b	II	*c*
Friesian	12	M	Jun 2009	B	b	II	*c*
Quarter horse	n. k.	Mc	Jun 2009	B	b	II	*c*
				
Warmblood	9	Mc	Aug 2008	A	a	III	*c*
				
English Thoroughbred	11	Mc	May 2005	D	r	IV	*d*
				
Warmblood	13	Mc	Jun 2007	S	l	III	*s*

^a ^age in years at the time of diagnosis; n. k., not known

^b^F, female; M, male; Mc, male castrated

^c ^See Table 1

### Phylogenetic analysis

**Figure 1:**
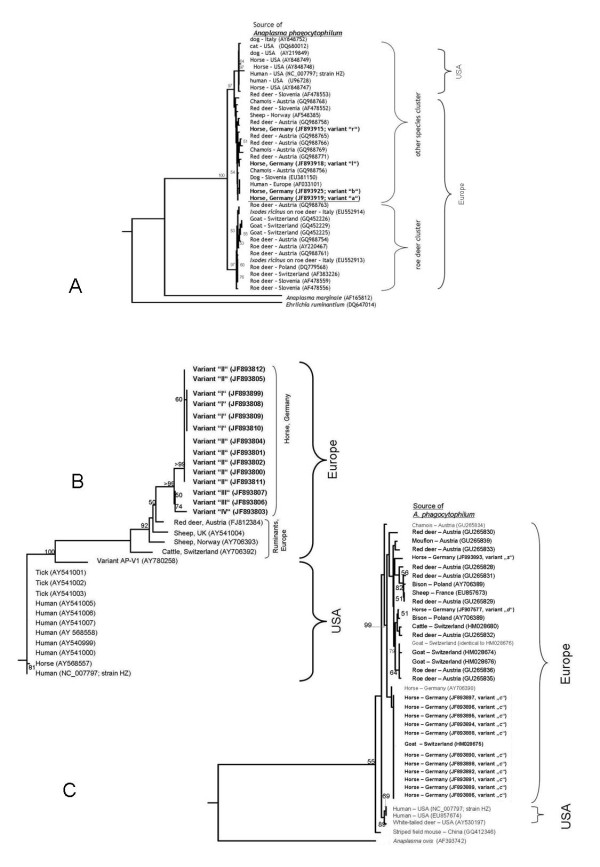
**Phylogenetic trees of 530 bp sequences of the *groEL *(A), 893 bp sequences of the *msp2 *(B) and 341 bp sequences of the *msp4 *genes (C)**. The phylogenetic trees include sequences obtained in this study from *Anaplasma phagocytophilum *from horses in Germany (this study: bold print) and selected *A. phagocytophilum *sequences available from GenBank. Bootstrap values >50% (1000 repeats) are presented at the respective nodes. GenBank accession numbers are indicated in parentheses, for sequences from this study the lettering used for the variants as found in Table 2 is indicated. The following gene sequences derived from GenBank [accession no.] were used in the construction of the phylogenetic trees. They are 100% identical in the analysed part to *A. phagocytophilum *GenBank sequences from the species indicated which were not included in the phylogenetic trees. Tree (A): [JF893915]: red deer - Slovenia; [GQ988765]: mouflon and chamois - Austria; [GQ988766]: Alpine ibex - Austria; [GQ988756]: cattle - Germany; [EU381150]: wild boar - Slovenia, horse - Sweden, Switzerland and Germany; [JF893925]: dog - Italy and Slovenia; [JF893919]: dog and roe deer - Slovenia; [DQ680012]: California wood rat, hispid cotton rat, gropher snake, and cottontail rabbit - USA; [AF548385]: cattle - Germany; Tree (C): [GU265829]: mouflon and chamois - Austria; [GU265831]: Alpine ibex - Austria; [AY706389]: cattle - France and Switzerland; [GU265832]: sheep; [HM028676]: roe deer - Spain; [JF893886]: sheep, goat - Switzerland.

Phylogenetic analysis of the partial *groEL *(530 bp), the partial *msp2 *(893 bp) and the partial *msp4 *(343 bp) genes was performed with sequences from this study and selected sequences from GenBank (Figure 1). As previously shown, the *groEL *tree clustered into *A. phagocytophilum *sequences from roe deer and those from other species. The horse sequences clustered within the "other species" group (Figure 1A). Interestingly, one of the partial groEL sequences from horses in this study (variant "a"; GenBank [JF893919]) has been detected previously in roe deer when comparing the amplified 530 bp, according to the GenBank entry [AF478558]. The *msp2 *phylogenetic tree showed clusters in sequences from US and from Europe; and within these clusters, ruminants clustered separately (Figure 1B). The *msp4 *phylogenetic tree showed that also US strains clustered separately from the European sequences. The variant "c" clustered separately from the others with a partial *msp4 *gene previously deposited in GenBank whereas the other two variants "*s*" and "*d*" clustered with sequences from ruminant species (Figure 1C).

## Discussion

For 14 cases of EGA the causing agent *A. phagocytophilum *was analysed with molecular methods for variations in four partial genes. Altogether, four different partial *16S rRNA *gene variants of *A. phagocytophilum *were detected to infect horses in Germany. The variant "B" was found identical to the "prototype" variant causing disease in humans in the amplified region [GenBank: U02521] and it was discovered in 11 horses.

This variant has previously been detected in dogs, horses and in roe deer, red deer and sheep [31,32,17,15,7,5]. The *16S rRNA *gene variant "B" seems rather rare in ticks in Germany whereas the most frequent variant in ticks seems to be "A" [3, 29, 33, Schorn S. unpublished data]. It was detected in a sample from one horse in this study. *16S rRNA *gene type "S" has been detected in sheep, red deer, roe deer, ibex and *I. ricinus *[7,3,5], whereas type "D" has not been detected yet. Previous studies could not show a host species segregation on basis of the *16S rRNA *gene [5,7]. However, by comparing data from our study to GenBank and to previous own investigations [6,7], it became evident that there seem to be *16S rRNA *gene variants infecting wild and domestic ruminants (e.g. "X","Y" (Table 1), as described in [6,7]), and others being the main variants causing disease in humans, horses and dogs (e.g. "B"; [30,32,34]). This could thus indicate potential different natural cycles, even when variants can not be attributed to a particular species. Two variants detected from the clinical EGA cases in this study ("B", "S") seem, however, not confined to domestic animals, but also appear in wild animals which might act as reservoirs (like red deer and roe deer), whereas the possibly "apathogenic" variants ("X", "Y", Table 1), seem to be specific for wild ruminant populations and possibly goats [6,7]. Several questions remain unanswered: may a reservoir animal or a tick form a "bridge" between a potential wild animal and domestic cycle, or could the infection of domestic animals be considered a "spill-over" of variants like "B" and "S"? I. e. are all variants circulating in tick-ruminant cycles, and only a small variety of those cause disease? Horses, dogs and humans have previously been proposed as accidental hosts [35].

The American variant Ap-V1 (matching "X" [6,7] in the amplified part of the *16S rRNA *gene), which is supposedly apathogenic, probably has the white-tailed deer as natural reservoir [36]. This variant seems not to cross to dogs, horses and humans either. Two fully different enzootic cycles in nature were suggested for the Western United States as there was no evidence for spatial, genetic or clinical overlap between *A. phagocytophilum *variants from granulocytic anaplasmosis cases (humans, horses and dogs) and those from the natural wildlife reservoir, the dusky-footed wood rat enzootic cycle [37].

The partial *groEL *gene variant "b" from this study has previously been detected in horses with EGA in Europe [15,19,38], but horses suffering from EGA from Italy (Sardinia) contained different variants, which were not detected in this study [28]. GroEL-variant "r" has previously been detected from red deer in Slovenia and "l" has been previously detected from an *I. ricinus *tick in Germany. GroEL-variant "a" has been detected from both dog and roe deer in Slovenia. The clustering of the *groEL *gene in phylogenetic analysis into a cluster containing sequences from roe deer and another containing sequences derived from other species may show different ancestry for different *A. phagocytophilum *variants. Some variants may have evolved in a natural cycle adapted to roe deer which may be separate from other variants involving other host species. However, the variant "a" has also been detected from roe deer, which seems to be exceptional when comparing the clustering of this and of previous studies [28]. This observation deserves more attention in future studies on genetic variants of *A. phagocytophilum*. Whereas the observed nucleotide substitutions in the partial *groEL *gene did not result in amino acid changes, the variation in the partial major surface protein 2 coding gene did. In the phylogenetic analysis, the partial *msp2 *gene showed strong clustering between the continents. This may be due to a possible reaction or adaptation to the different vector or host species in the different geographic locations, as it may reflect different host immunity and an adaptation of the pathogen to it [39]. On the other hand, it may be that host specificity or susceptibility are more important than geographic origin. In an experimental study on cross transmission in the US, host susceptibility and transmissibility of *A. phagocytophilum *may have played a more important role than the vector tick itself [40], which suggests different reservoir host cycles.

The amplification of the partial *msp4 *gene showed little heterogeneity among horses, both on the nucleotide and at the amino acid level, whereas in large wild animals it showed great heterogeneity, also on amino acid level [7]. In a previous study, the partial *msp4 *gene variants from ruminants and those from dogs, horses and humans clustered separately [30]. It was suggested that *A. phagocytophilum *from ruminants may share characteristics, like reservoir and pathogenicity, and that they may be different from the variants found in humans. This strengthens the hypothesis that different enzootic cycles or niches of different variants possibly involving different reservoir hosts exist in nature. A thorough study of the *ankA *gene from different animal sources came to similar conclusions [5,34]. It was hypothesised that ruminant species can host a larger variety of *A. phagocytophilum *isolates due to a possible co-evolution of host and pathogen [34]. However, we detected two variants in the partial msp4 gene that clustered within the ruminant cluster. This raises the question whether these two cases in the horses may represent a "spill-over" from ruminant variants, or whether the clustering of the *msp4 *gene phylogenetic tree may only be weakly supported due to the relatively short sequence used for the comparative analysis. The use of *16S rRNA *gene or parts of it as a tool for the molecular characterisation of *A. phagocytophilum *has been criticized [4,5,32]. Nonetheless the *16S rRNA *gene may be very useful, on a broader level, for discriminating variants eliciting granulocytic anaplasmosis from those from a potential wild ruminant cycle. Even though the true genetic variety may be much larger as has been shown e.g. for *ankA *and *msp2 *([3]; results from this study) and a more comprehensive analysis of genetic variants is necessary, the *16S rRNA *gene typing seems a good method for initial typing.

## Conclusions

We conclude that more than one genetic variant of *A. phagocytophilum *infects horses and causes EGA in Germany and possibly elsewhere. One main causing variant was 100% similar in the amplified part of the partial *16S rRNA *gene to the one pathogenic for humans [GenBank: U02521], but other variants caused clinically apparent infection, too. Comparing this data to sequence data from ticks another type ("A") seems to be the most prevalent one in ticks, but seems to be rather rare in causing disease in horses [[29], Schorn et al., unpublished data]. This gives a strong basis for hypothesising that *A. phagocytophilum *may exist in different natural cycles involving different reservoir hosts. This tendency has also been shown in previous studies [5,30]. We further conclude that for a preliminary comparative typing of *A. phagocytophilum *the partial *16S rRNA *gene seems a useful tool. Therefore, even when other genes are studied for phylogenetic or other differentiation or in the search for better markers for typing, *16S rRNA *variant typing should also be performed.

## Methods

### Horses and clinical assessment

**Figure 2:**
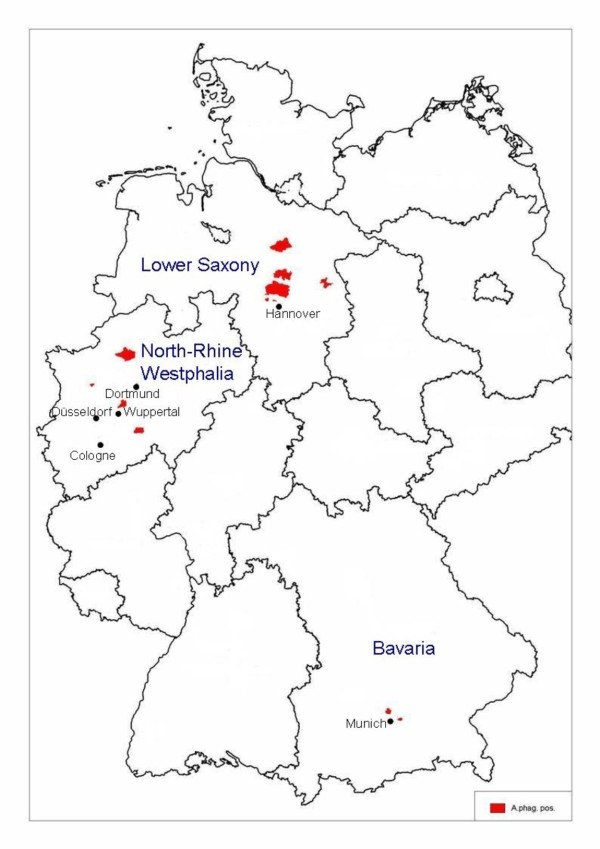
**Map of Germany showing the origin of the horses included in this study**. The location where the horses were staying at the time of diagnosis is shown according to the postal code in the respective districts (shaded in red) within the federal states. The map was created with Regiograph (GfK MACON AG, Waghäusel, Germany).

Fourteen cases of clinical EGA, diagnosed from 2004 to 2009, were included in this study (Table 2). The horses were from three federal states of Germany: Lower Saxony (n = 6), North-Rhine Westphalia (n = 6) and Bavaria (n = 2) (Figure 2). The diagnosis which had been requested by the veterinarians for routine diagnostics at the time of infection had been taken with Giemsa stained buffy coat smears. In all 14 horses, morulae had been detected in the granulocytes. For 3 horses, diagnosis at the time had also been taken by PCR. EDTA-blood which was left over from the routine diagnosis of the horses was preserved at -20°C for further use in this study. All 14 animals were suffering from high fever, 8 of them recurring, 5 horses had icterus, 2 a lower limb oedema, and further 2 anorexia. 13 animals showed thrombocytopenia, 6 monocytopenia, and 5 lymphopenia, the total bilirubin was increased for 5 horses. 7 horses were previously treated with penicillin or other antibiotics not acting against *A. phagocytophilum*.

### DNA-Extraction and Polymerase Chain Reaction

DNA was extracted with the HighPure PCR Template Preparation Kit (Roche, Mannheim, Germany). The presence of specific *A. phagocytophilum *DNA was verified in all samples with a real-time PCR targeting the *msp2 *gene as previously described ([41,6]). The conditions and primers for sequence analysis of the partial *16S rRNA*, *groEL*, *msp2*, and *msp4 *genes are listed in Table 3. The PCRs were carried out on the thermocyclers GeneAmp System 2700/Veriti (Applied Biosystems, Weiterstadt, Germany) or an Eppendorf Mastercycler gradient (Eppendorf, Hamburg, Germany). The HotStarTaq Kit (Qiagen, Hilden, Germany) was used for all PCR experiments. Positive (DNA of *A. phagocytophilum *infected *I. ricinus *ticks) and negative (molecular biology grade water) controls were included in each PCR run. Successful amplification of the gene targets was verified with 2.0% agarose gel-electrophoresis after staining with GelRed^® ^(Biotium, Hayward, USA) and visualized under UV-light.

**Table 3:** Primers used for the PCRs for sequence analysis for *Anaplasma phagocytophilum *from horses in this study.

Target Gene	Primers 5'-3'	Cycle conditions **	Reference
*16S rRNA*	First reaction: ge3a: CACATGCAAGTCGAACGGATTATTC ge10r: TTCCGTTAAGAAGGATCTAATCTCC Nested reaction*: ge9f: AACGGATTATTCTTTATAGCTTGCT ge2: GGCAGTATTAAAAGCAGCTCCAGG	40 cycles (nested reaction: 25 cycles): 30 sec 94°C, 30 sec 55°C, 1 min 72°C	[42]

*groEL*	First reaction: EphplgroEL-F: ATGGTATGCAGTTTGATCGC EphplgroEL-R: TCTACTCTGTCTTTGCGTTC Nested reaction*: EphplgroEL-F: ATGGTATGCAGTTTGATCGC EphgroEL-R: TTGAGTACAGCAACACCACCGGAA	40 cycles: 30 sec 94°C, 30 sec 55°C, 45 sec 72°C	[28]

*Msp2*	msp25: TTATGATTAGGCCTTTGGGCATG* msp23:TCAGAAAGATACACGTGCGCCC*	35 cycles: 1 min 95°C, 1 min 62°C, 1.5 min 72°C	[43]

*Msp4*	First reaction: MSP4AP5: ATGAATTACAGAGAATTGCTTGTAGG MSP4AP3: TTAATTGAAAGCAAATCTTGCTCCTATG	40 cycles: 30 sec 94°C, 45 sec 54°C, 1 min 72°C	[30]
	Nested reaction*: msp4f: CTATTGGYGGNGCYAGAGT msp4r: GTTCATCGAAAATTCCGTGGTA		[44]

* Primers used for the sequencing reactions

** all PCR reactions: 15 min 95°C initial activation; 7 min 72°C final extension

### Sequence Analysis

PCR products were purified with the QIAquick PCR Purification Kit according to manufacturer's instruction (Qiagen, Hilden, Germany) and sent for sequencing with forward and reverse primers (Table 3) to Eurofins MWG Operon (Martinsried, Germany). The obtained chromatograms were evaluated with Chromas^©^Lite http://www.technelysium.com.au/chromas_lite.html. ClustalW2 was used to perform multiple sequence alignment of the sequences [45]. Nucleotide sequences were translated to amino acid level using Transeq http://www.ebi.ac.uk/emboss/transeq/. Sequence homology searches were made by BLASTn analysis of GenBank http://www.ncbi.nlm.nih.gov.library.vu.edu.au/BLAST/.

### Phylogenetic analysis

Phylogenetic analysis was carried out with the PHYLIP 3.67 software package [46] using the neighbor joining method. For this analysis 33 sequences of the partial *groEL *gene, 16 of the partial *msp2 *gene and 22 of the partial *msp4 *gene were used from GenBank. Distance matrices were calculated with the Kimura 2-parameter method using DNADIST. A bootstrap analysis was performed to test the stability of the trees with 1000 resamplings using SEQBOOT. Consensus trees were derived with CONSENSE.

## Competing interests

The authors declare that they have no competing interests.

## Authors' contributions

CS organised and established the laboratory work, carried out the sequence and phylogenetic analysis and wrote the manuscript. GL provided the samples and clinical data for this study, and GL and KP critically revised the manuscript. All authors read and approved the final manuscript.
